# Editorial

**Published:** 1840-12-12

**Authors:** 


### THE MEDICAL EXAMINER.

PHILADELPHIA, DECEMBER 12, 1840.

### TO SUBSCRIBERS.

With the approaching termination of the year will be concluded the third volume of the Medical Examiner. From this date, one of the present editors, Dr. Clymer, still in Europe, retires, and, from the commencement of the fourth and succeeding volume, Dr. W. Poyntell Johnston will be associated with Drs. Biddle and Gerhard in the editorial duties of the journal. The prolonged absence of one of the editors in Europe, during which he has been prevented from contributing actively to the columns of the Examiner, with other circumstances, has occasioned an unpunctuality in the publication of the journal, and a want of variety in the amount of original matter, which future arrangements will, it is hoped, correct. Hereafter, the editors pledge themselves that the Examiner shall be issued without fail on the Saturday of each week. A corresponding improvement in its character they confidently hope to effect. The large and constantly increasing circulation of the Examiner, is a gratifying proof that their efforts to publish an independent and useful medical journal have not been hitherto without success. These efforts, for a time relaxed from the press of circumstances, they will be enabled for the future to pursue with strengthened hands and renewed vigour.

Edward Peace, M. D., has been elected one of the Surgeons of the Pennsylvania Hospital, in the place of Thomas Harris, M. D., resigned. Dr. Harris vacates a post which he has filled for twelve years with distinguished success, enjoying the entire confidence and respect of the profession, the public, and the managers of the institution. General regret is felt at his determination to retire from the active duties of the hospital, to the discharge of which he brought a rare combination of experience, judgment, operative skill, and talent as a teacher.

The Surgeons of the Pennsylvania Hospital now are:—Jacob Randolph, M. D., George W. Norris, M. D., and Edward Peace, M. D. Dr. Randolph has just sailed in the Great Western for Europe, where he proposes to pass a year or two.

The physicians to the hospital are B. H. Coates, M. D., Geo. B. Wood, M. D., and T. Stewardson, M. D.

Thomas S. Kirkbride, M. D., is the physician to the Insane Department of the hospital, situate in the district of Blockley, on the western bank of the Schuylkill. He will shortly enter upon the duties of this appointment, residing in the institution.

